# Plant-Based Diet Quality Is Associated with Cardiometabolic Health in Adults: A Cross-Sectional Analysis of the Australian Health Survey

**DOI:** 10.3390/nu17101621

**Published:** 2025-05-09

**Authors:** Kacie M. Dickinson, Laura E. Marchese, Katherine M. Livingstone

**Affiliations:** 1 Caring Futures Institute, Nutrition and Dietetics, Flinders University, Adelaide 5042, Australia; kacie.dickinson@flinders.edu.au; 2 Institute for Physical Activity and Nutrition (IPAN), School of Exercise and Nutrition Sciences, Deakin University, Melbourne 3125, Australia; k.livingstone@deakin.edu.au

**Keywords:** plant-based diet quality, cardiometabolic health, blood pressure, obesity, adults

## Abstract

**Background/Objectives:** Evidence suggests that plant-based dietary patterns are beneficial for cardiometabolic health. However, it is unclear whether the quality of plant-based dietary patterns is differentially associated with cardiometabolic health. This study aimed to examine the association between three plant-based diet quality indices and cardiometabolic health among Australian adults. **Methods:** Data on 4877 adults (mean 45.1 years) from the cross-sectional Australian Health Survey 2011–2013 were used. Three plant-based diet quality indices (overall, healthful, and unhealthful) were derived from two 24 h dietary recalls. Anthropometric measures and blood pressure were assessed by trained interviewers. High-risk waist circumference was classified as ≥80 cm in females and ≥80 cm in males. Hypertension was defined as >140/90 mmHg. Fasting blood samples were used to estimate lipid profiles and diabetes status (based on plasma glucose or HbA1c). Multivariate logistic regression analyses were used to investigate the association between plant-based diet indices and cardiometabolic markers. **Results:** The healthy plant-based diet index was associated with lower odds of a high-risk waist circumference (OR 0.98; 95%CI 0.96–0.99), and the unhealthy plant-based diet index was associated with increased odds of having hypertension (OR 1.02; 95%CI 1.00–1.04). No other significant associations were identified between the plant-based diet quality indices and cardiometabolic outcomes. **Conclusions**: There was some evidence that the quality of plant-based dietary patterns was differentially associated with cardiometabolic health, with healthier plant-based dietary patterns associated with lower odds of a high-risk waist circumference, and less healthy plant-based dietary patterns associated with increased odds of hypertension. These findings support the consideration of the quality of plant foods consumed, and the need for targeted advice for optimising cardiometabolic health among adults.

## 1. Introduction

The diet is a leading risk factor for non-communicable disease [1,2]. In particular, evidence supports plant-based diets for optimising cardiometabolic health and the prevention of cardiovascular disease (CVD) [1,3,4,5,6,7,8]. Conversely, research suggests that diets that are high in saturated fat, processed meats, and sodium are associated with a higher risk of CVD [9]. The exact mechanisms for the beneficial effects of plant-based diets on non-communicable diseases are unclear; however, evidence suggests that key mechanisms such as hypertension, diabetes, inflammatory markers, and weight may contribute to disease progression [6,7,8].

Plant-based diets can be defined as consuming foods predominately derived from plants, whilst minimising animal food products [10]. This is distinct from vegetarian and vegan diets, which exclude meat or animal-derived products. Plant-based diets should encourage high intakes of whole foods aligned with dietary guidelines [11], such as whole vegetables, fruits, legumes, and grains. However, discretionary foods such as chips, confectionary and sugar sweetened beverages, which are often high in energy, fat, sugar and sodium, are also plant-based foods. Thus, plant-based diet patterns adopted by the population may not always facilitate healthy food choices and diets.

Given that food and nutrients are not consumed in isolation, increasing research is investigating overall dietary patterns, or diet quality [12]. Diet quality indices, such as the Dietary Guideline Index (DGI), have been used to examine how dietary intakes compare to national recommendations [13,14,15]. Similarly, diet quality indices have been derived to reflect adherence to plant-based diets [16]. Previous research has focused on plant-based diet types, such as vegetarian and vegan diets [17]; however, emerging research has derived plant-based diet indices that reflect the quality of the components, such as the level of processing [8,18,19,20].

The overall, healthy and unhealthy plant-based diet indices have been applied globally to understand associations with markers of cardiometabolic health [6,7,8,16,21]. However, limited research has examined these indices using nationally representative data on Australian adults [22]. Understanding plant-based diets and their relationship with diet quality and cardiometabolic health will inform policies, interventions, and clinical practice that are targeted to healthier plant-based foods. Therefore, this study aimed to examine the association of three plant- based diet indices (overall, healthful and unhealthful) with a range of cardiometabolic health markers in Australian adults.

## 2. Materials and Methods

### 2.1. Study Population

**Figure 1:**
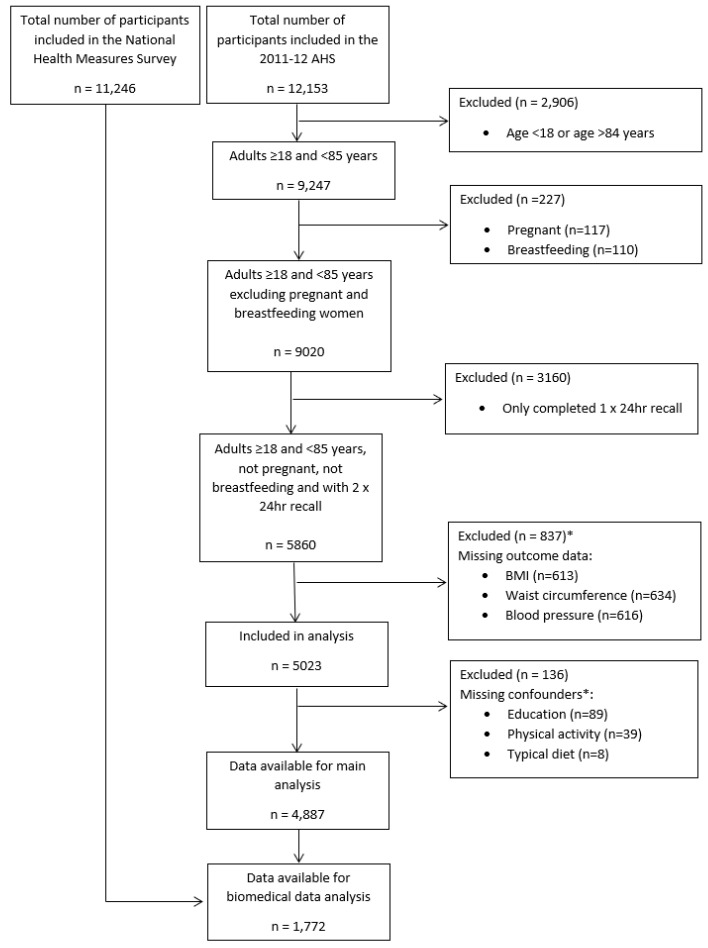
Flow diagram of participants included in the analysis of the 2011–13 AHS * categories are not mutually exclusive.

The current study has drawn upon data collected as part of the Australian Health Survey (AHS) (2011–2013), which is the largest and most comprehensive health survey conducted in Australia. Methods and sampling procedures have been described in detail elsewhere [23,24]. The data used were collected during the National Nutrition and Physical Activity Survey (NNPAS) (2011–2012) and National Health Measures Survey (2011–2013), two components of the AHS. Of the 30,329 respondents from the NNPAS, 11,246 participated in the National Health Measures Survey to provide blood and urine samples (response rate: 37.1%). Inclusion criteria were males and females aged 18–84 years for whom measured height and weight were reported and that had completed two 24 h dietary recalls. Participants were excluded if they were (1) pregnant or breastfeeding; (2) had missing data for any outcomes, exposures, or covariates specified, or (3) only had 1 day of dietary recall (Figure 1). Characteristics of people excluded compared with the analytic sample (*n* = 4887) are presented in the Supplementary Table S2. A total of 1772 participants had biomedical data available from the National Health Measures Survey for which they also had two 24 h diet recall diet data. The Strengthening the Reporting of Observational Studies in Epidemiology-nutritional epidemiology (STROBE-nut) statement (Supplemental Table S1) was used to report this study [25].

### 2.2. Dietary Intake Assessment

Data from the two 24 h dietary recalls conducted at the face-to-face interview and then follow-up telephone interview were used to estimate nutrient and food intakes in the present analysis [23,24]. The 24 h dietary recall was conducted by trained interviewers using a computer assisted interview system, the Automated Multiple-Pass Method, to ensure consistent and complete data collection. A second 24 h dietary recall, via telephone interview, was collected at least 8 days after the first interview (response rate: 63.6%). Only participants who completed both recalls were included in the present analysis. Nutrient intakes were estimated from the AUSNUT 2011–2013 Food Nutrient Database [26].

### 2.3. Plant-Based Diet Quality

Using the NNPAS diet intake data we derived three different plant-based diet scores to differentiate between quality of plant-based food intake drawing on previously established methodologies by Satija et al. [27]. These are: an overall plant-based diet index (PDI), a healthful plant-based-diet index (hPDI) and an unhealthful plant-based diet index (uPDI). As current methods are based on food frequency questionnaire data, we adapted these methods used for 24 h recall data by classifying foods consumed (in grams) at the 3-, 5- and 8-digit level from the food composition database (AUSNUT 2011–2013) that was developed for the NNPAS [26]. Food items in the AUSNUT databases are identified using unique eight-digit codes using nested hierarchical food group coding; the first two digits indicate the major food group, followed by three digits for the sub-major food group and the remaining five digits for the minor food group. These foods were classified into 18 food groups based on similarities in nutrients and culinary purpose within the larger categories of healthy plant foods (wholegrains, fruits, vegetables, nuts, legumes, vegetables oil, tea/coffee); less healthy plant foods (fruit juices, refined grains, potatoes, sugar-sweetened beverages, sweets/desserts); and animal foods (animal fat, dairy, eggs, fish/seafood, meats, mixed dishes, where meat is an ingredient). In previous research [27] margarine was excluded from the PDI as the fatty-acid composition of margarines in the US has changed. However, we included margarine in PDI given most industrial trans-fats have been removed from margarine in Australia since 1996 [28].

Supplementary Table S3 contains the list of all major and sub-major food groups in AUSNUT and their corresponding classification to one of eighteen food groups according to previously published methods [27]. The Multiple Source Method (MSM) software was used to estimate usual intake distributions within each food group (using agec, sex, and age * sex interaction term as covariates) [29].

The intake of each food group (in grams) was ranked into consumption quintiles and given positive or reverse scores. The PDI was created by giving positive scores to all plant food groups and reverse scores to all animal food groups. For positive scoring, participants who fall above the highest quintile of a food group will receive 5, through to participants below the lowest quintile for a food group who will receive a score of 1. For reverse scores, those above the highest quintile will receive a score of 1 and those below the lowest quintile a score of 5. The scores from the 18 food groups are then summed to generate a possible overall score between 18 and 90. For the hPDI, healthy plant-based food groups were scored positively and unhealthy plant food groups and animal food groups negatively. For the uPDI, foods in the less healthy plant food group will be given positive score and foods in the health plant food group and animal food group will be given reverse scores. All plant-based diet quality index scores were normally distributed.

### 2.4. Outcomes

#### 2.4.1. Body Mass Index and Waist Circumference

Body mass index (BMI) and waist circumference were measured using standardised protocols and have been previously described [14,23,24]. Briefly, trained interviewers assessed weight (kg), height (cm), and waist circumference (cm) measurements on a voluntary basis using digital scales, a stadiometer, and a metal tape, respectively. Height and weight were used to calculate BMI (kg/m^2^) using Quetelet’s metric, and standard cut-offs were used for BMI and waist circumference categories [30]. High risk waist circumference was defined as ≥80 cm for females (<80 not at risk) and ≥94 cm for males (<94 cm not at risk).

#### 2.4.2. Blood Pressure

Participants had systolic and diastolic blood pressure measured on the left arm using an automated blood pressure monitor on two consecutive occasions by trained ABS staff. As detailed elsewhere [23,24], a third measurement was taken in cases where the systolic and diastolic pressures differed more than 10 mmHg. When only two readings were needed, the second reading was used for systolic and diastolic pressure measures. If a third reading was needed, the average of the second and third readings was used. Participants were classified as having high blood pressure using two definitions [31,32]. The first was based on the Australian guidelines for hypertension: systolic blood pressure ≥ 140 mmHg and diastolic blood pressure ≥ 90 mmHg; non-hypertensive was defined as <140/90 mmHg [31]. The second was based on the US guidelines for high blood pressure: systolic blood pressure > 120 mmHg and diastolic blood pressure > 80 mmHg; normal blood pressure was defined as <120/80 mmHg [32].

#### 2.4.3. Lipid Profiles and Diabetes Status

The methods for obtaining blood samples have been previously described in detail elsewhere, with collection and analysis following quality control and quality assurance procedures [14,23,24]. Briefly, urine and blood samples were collected at pathology collection clinic or via a home visit using standard operating procedures for assessment of total blood cholesterol, HDL-cholesterol and LDL-cholesterol (mmol/L), triglycerides (mmol/L), blood glucose (mmol/L), and Hb A1c (HbA1c) (%). Results for LDL-cholesterol, triglycerides, and plasma glucose were obtained from participants who had fasted for at least 8 h. Total cholesterol, HDL cholesterol, and HbA1c were measured without the need for fasting. Participants’ use of lipid lowering medication was not recorded. Total cholesterol status was classified as normal (total cholesterol < 5.5 mmol/L) or abnormal cholesterol status (≥5.5 mmol/L). For HDL-cholesterol status, normal was defined as ≥1.0 mmol/L in males and ≥1.3 mmol/L in females. Abnormal LDL-cholesterol was defined as ≥3.5 mmol/L (persons with fasting triglycerides ≥ 4.5 mmol/L excluded). Abnormal triglycerides status was defined as ≥2.0 mmol/L. Participants were classified as not having dyslipidaemia if they had ‘Normal’ cholesterol, HDL (sex-dependent), LDL and triglyceride levels. Diabetes status was determined using two criteria that were derived from fasting blood samples on the day of the survey (1) self-reported diabetes, impaired fasting glucose > 6.0 mmol/L or fasting plasma glucose ≤6.0 mmol/L indicating no diabetes (2) self-reported or at risk of diabetes HbA1c ≥ 6.0%.

### 2.5. Covariate Assessment

Covariates considered were based on the previous literature and via a directed acyclic graph (DAG) (Supplementary Figure S1). Socio-demographic characteristics were collected in the NNPAS via interviewer-administered questionnaires. Age was used as a continuous variables and sex was defined as female or male. Smoking status was defined as current smoker, ex-smoker, or never smoked [14]. Physical activity was categorised as meeting current guidelines (150 min over 5 sessions per week) or not [23]. Education status was classified as no non-school qualification, certificate/diploma, or tertiary qualification. Typical dietary intake information was collected on the day of reporting and was classified as (more than usual, usual, and less than usual). Energy misreporting was calculated as the ratio of energy intake to predicted total energy expenditure (using sex and age-specific equations based on body weight (kg) and a physical activity level (PAL) of “low active” (PAL ≥ 1.4 < 1.6) [33]. Participants’ energy intake from NNPAS was classified as plausible, under-reporting, or over-reporting. Alcohol intake (wine consumer or non-consumer) was also included as covariates given bi-directional relationship of alcohol with CVD risk.

### 2.6. Data Access and Ethical Considerations

The Census and Statistics Act 1905 provides ethics approval for the Australian Bureau of Statistics to conduct the household interview components of health surveys. Ethics approval for the NHMS was granted by the Australian Government Department of Health and Ageing Departmental Ethics Committee. Permission was obtained from the Australian Bureau of Statistics to access the basic confidentialised unit record file, released on 3 June 2015, to enable data analysis.

### 2.7. Statistical Analysis

Complete case analysis was used. A description of missing data is in Figure 1. Since NNPAS 2011–2012 was conducted using a stratified multistage area sampling of private dwellings, sampling weights were applied when producing estimates to compensate for unequal probability of selection of the sampled person, non-response, and non-coverage. Multivariate logistic regression analyses were used to investigate the association between plant-based diet scores (independent variable) and cardiometabolic risk factors (dependent variables). Analyses were adjusted for age (continuous), sex, smoking status (categorical: current smoker, ex-smoker, never smoked), physical activity (categorical: Met or not met 150 min 5 sessions per week guideline), education (categorical: no non-school qualification, certificate/diploma, tertiary), typical diet (categorical: more than usual, usual, less than usual), energy misreporting (plausible, under-reporting, over-reporting), alcohol intake (categorical: non-consumer, consumer) and BMI (continuous; not included in analysis of anthropometric outcomes). Survey weighting calibrated against population benchmarks (i.e., age, sex, and area of usual residence) were used to account for the complex survey design. These weightings were specifically designed by the Australian Bureau of Statistics to account for bias associated with those who volunteered to participate in the second dietary interview (default used for all analyses) and for the assessment of biomedical outcomes (used for diabetes and lipid data). *p* < 0.05 was considered statistically significant. Analyses were undertaken using STATA (Version 15 Stata Corp., College Station, TX, USA).

## 3. Results

There were 4877 participants included in the primary analysis (female = 2542; male = 2345) (Figure 1). A comparison of characteristics of those included and excluded from the analysis is presented in Supplementary Table S2. The participants excluded from the analysis were mostly similar to those included in the analytic sample across all characteristics. Participant characteristics according to tertiles of plant-based diet quality are presented in Table 1. Among the highest tertile of hPDI there was a higher proportion of adults who were older, female, more highly educated, and of a healthy body weight compared to the lowest tertile of hPDI. Among the highest tertile of uPDI there were a higher proportion of adults who were younger, male, and had lower proportion of higher education qualifications compared with the lowest tertile of uPDI.

### 3.1. Plant-Based Diet Patterns and Outcomes

#### 3.1.1. Weight Status, Waist Circumference, and Blood Pressure

A total of 61.7% of the sample had overweight/obesity and 61.9% had a waist circumference that was classified as increased risk, 57.4% had high blood pressure (>120/80 mmHg), and 20.8% were hypertensive (>140/90 mmHg; Table 2).

There were no significant associations between the three diet quality indices and odds of overweight or obesity, with or without adjustment for confounders. In unadjusted models, the hPDI was associated with a higher odds of an at-risk waist circumference (OR 1.03; 95%CI 1.02–1.05); after adjusting for confounders, hPDI was associated with lower odds (OR 0.98; 95%CI 0.96–0.99). uPDI was associated with lower odds of an at-risk waist circumference (OR 0.96; 95%CI 0.95–0.97); after adjusting for confounders, this was no longer significant. There was a negative association between the PDI and the uPDI, and high blood pressure and hypertension; however, after adjustment for confounders these were attenuated and only the uPDI was associated with higher odds of hypertension (OR 1.02; 95%CI 1.00–1.04). hPDI was associated with higher odds of high hypertension (OR 1.02; 95%CI 1.00–1.03); however, this was no longer significant after adjusting for confounders.

#### 3.1.2. Lipids and Glycaemic Control

Of the 4877 participants with data on obesity, waist circumference, and blood pressure, 1772 participants also had biomedical data. Of these, 33.6% had high total cholesterol, 20.7% had low HDL cholesterol, 34.9% had high LDL cholesterol, 11.1% had high triglycerides, 54.8% had dyslipidaemia, 3.6% had impaired fasting plasma glucose, and 5% had HbA1C levels that indicated presence of or at risk of diabetes (Table 3). hPDI was associated with higher odds of dyslipidaemia (OR 1.03; 95%CI 1.00–1.05), while uPDI was associated with lower odds of dyslipidaemia (OR 0.95; 95%CI 0.93–0.97). hPDI (OR 1.04; 95%CI 1.02–1.06) and uPDI (OR 0.94 (95%CI 0.92–0.97) were associated with total cholesterol, but after adjusting for covariates these associations were no longer significant. Similarly, hPDI (OR 1.03; 95%CI 1.01–1.05) and uPDI (OR 0.95; 95%CI 0.93–0.97) were associated with high LDL cholesterol, but after adjusting for covariates these associations were no longer significant. The hPDI was associated with higher odds (OR 1.03; 95%CI 1.00–1.07) and uPDI was associated with lower odds (OR 0.96; 95%CI 0.93–0.99) of having or being at risk of diabetes based on HbA1c; however, this was no longer significant after adjusting for confounders. There were no significant associations between plant-based diet indices and HDL, triglyceride status, and diabetes status based on fasting plasma glucose (Table 3).

## 4. Discussion

In the current study, we aimed to explore the association between three plant-based diet indices and cardiometabolic health in a representative sample of Australian adults. The key findings were that a higher healthy plant-based diet pattern (hPDI) was associated with lower odds of a high-risk waist circumference and that a higher unhealthy plant-based diet pattern (uPDI) was associated with higher odds of hypertension. This study adds new evidence of these relationships in a nationally representative sample of Australian adults that can be used for population monitoring and surveillance. These results highlight the beneficial role of a diet high in minimally processed plant-based foods, such as wholegrains, fruits, vegetables, nuts and legumes, on markers of adiposity and the detrimental role of a diet high in processing plant-based foods, such as refined grains, sugar-sweetened beverages, and confectionary, on blood pressure. Findings from this study emphasise the importance of the healthiness of the foods in a plant-based diet, and that a plant-based diet alone may not be sufficient for the prevention of poor cardiometabolic health.

Consistent with previous research [34,35,36,37,38], following a healthy plant-based diet was associated with a more favourable waist circumference. Associations between vegan diets and a reduced waist circumference compared to omnivorous diets have been well reported in observational research [34]. Additionally, a recent umbrella review on the effect of plant-based diets on anthropometric and cardiometabolic markers in adults identified significant beneficial effects on waist circumference; however, this review did not focus on research using a dietary patterns approach, nor was the healthiness of the diet considered in the analysis [39]. A prospective cohort study of US adults found that participants who improved their diet over 20 years in alignment with the a priori diet quality score, a plant-focussed diet quality index similar to the one used in this study, had a reduced waist circumference over this time period [40]. Other cohort studies have also found that greater adherence to plant-based diet quality indices were also associated with a reduced waist circumference [35,36,37,38]. However, this study provided further insight into understanding this relationship by highlighting the importance of healthy plant-foods, rather than an overall effect on waist circumference just from reduced intake of animal foods. The exact mechanisms for this may be through consumption of ultra-processed foods, regardless of their plant or animal source [41].

This study found that an unhealthy plant-based diet was associated with higher odds of hypertension. This aligns with research internationally, where a prospective cohort study of 8041 mid aged US adults identified that those who highly adhered to an unhealthy plant-based diet had a 13% higher risk of hypertension [42]. Results from a prospective cohort study of 74,522 French women identified that a one standard deviation increase in the unhealthy plant-based diet index was associated with a 4% increase in hypertension risk [43]. Further to this, a South Korean community-based cohort of 5636 people identified that those with higher adherence to an unhealthy plant-based diet had a 44% higher incidence of hypertension [44]. Other plant-rich diets, such as DASH, Mediterranean, and portfolio diets also have convincing evidence of blood pressure lowering effects among adults with or at risk of hypertension [45,46,47,48]. Additionally, diets high in red meat have been shown to increase blood pressure in experimental studies. [49]. Thus, these results suggest that diets that are higher in unhealthy plant foods (such as sugar, sweetened beverages, and crisps) as well as low in animal foods increase the odds of higher blood pressure. Given the subcomponents contributing to the scoring of the uPDI, the results from this study are not unexpected. However, although the associations with high blood pressure measured with a lower cut-off (120/80 mmHg) were comparable, these did not reach statistical significance. Mechanisms for this relationship could be higher meat intake, as well as lower consumption of fruits, vegetables, and whole grains. Mixed dishes were also frequently positively scored on the uPDI and mainly comprise ultra-processed foods, which highlights a higher proportion of concerning nutrients such as sodium, saturated fat, and sugar. Future work is needed to explore human interventions of healthy and unhealthy plant-based diets, including ultra-processed plant foods, to elucidate mechanisms of this effect.

There was no observed relationship between plant-based diet indices and lipid profiles or glycaemic control. The evidence for these aspects of cardiometabolic health are mixed [50]. A review of 43 observational and intervention studies which included both traditional definitions of plant-based diets (such as vegan), and plant-based diet quality indices found that plant-based diets were associated with beneficial measures for lipid and lipoprotein profiles, using measures for decreased total cholesterol, low-density lipoprotein cholesterol, and apolipoprotein B concentrations [50]. However, effect sizes were greatest for plant-based dietary interventions, and there were less consistent associations found in observational studies using plant-based diet quality indices. Additionally, a recent systematic review and dose–response meta-analysis of 16 prospective studies using the same plant-based diet quality indices as the present study, identified that greater adherence to the PDI and hPDI were associated with a lower risk of type 2 diabetes, and the hPDI was associated with a higher risk [51]. The findings are in line with previous systematic reviews, meta-analyses, and umbrella reviews [39,52,53,54,55]. The lack of observed relationships in the present study is likely due to the small number of participants classified as having type 2 diabetes (*n* < 100), limiting statistical power to identify associations.

A strength of this study was the use of the Australian Health Survey, a nationally representative sample of adults. Survey weightings were used to account for non-response bias, and as shown in (Supplementary Table S2), the omitted sample were largely comparable to the sample included in the primary analyses. The MSM was used to estimate the usual dietary intake from the NNPAS. In the current study, we derived plant-based diet quality scores using dietary intake data from two 24 h recalls instead of food frequency questionnaires, which are used in the majority of the other papers published on this topic. The MSM provides greater precision in the classification of plant and animal foods given that ingredient level data are available. Most previous studies have used FFQ, which cannot distinguish animal/plant content of many mixed dishes in particular.

This study also has some limitations. The current study design is cross-sectional; therefore, causality cannot be determined. Some outcomes were self-reported, thus there is potential for reverse causality, as participants may have modified their diet in response to a previous diagnosis. The exclusion of people with missing outcome data may have also introduced selection bias. Confidentialised Unit Record File data access is limited to categorical outcome data; therefore, the analysis had a lower statistical power and level of precision compared to continuous outcome data [56]. The food composition database used to construct the plant-based diet quality indices (AUSNUT) is from 2011 to 2013, so does not represent the current plant-based food supply, where plant-based products are more readily available and consumed among the population [57,58].

The results from this study can be used to inform future interventions examining plant-based diets in modifying cardiometabolic risk factors among adults. Further population based and longitudinal studies are necessary to better understand the relationship between plant-based diets and cardiometabolic health trajectories across the lifespan given these diet patterns are emerging and being adopted more rapidly among some age and gender groups. As no association was found with BMI, these findings warrant future investigation with more precise measures of body composition, which can differentiate between fat mass and fat free mass.

## 5. Conclusions

This cross-sectional study of Australian adults showed some evidence that healthier plant-based diets were associated with more favourable anthropometric makers, while unhealthy plant-based diets were associated with higher blood pressure. These results highlight the beneficial role of a diet high in minimally processed plant-based foods on cardiometabolic health and the detrimental role of a diet high in processing plant-based foods. As these plant-based dietary patterns are increasingly adopted among the population, continued monitoring and surveillance of novel and ultra-processed plant-based foods and associations with cardiometabolic health should be a priority. Further, human interventions are needed to further understand the impact of these foods on cardiometabolic health and explore potential mechanisms by which they differ.

## Figures and Tables

**Table 1:** Participants’ characteristics according to tertiles of the three plant-based diet quality indices (PDI, hPDI, and uPDI).

	PDI			hPDI			uPDI			All
	T1 (*n* = 1845)	T2 (*n* = 1561)	T3 (*n* = 1481)	T1 (*n* = 1731)	T2 (*n* = 1689)	T3 (*n* = 1467)	T1 (*n* = 1705)	T2 (*n* = 1738)	T3 (*n* = 1444)	(*n* = 4887)
Age, years, mean (95%CI)	45.2 (44.2, 46.3)	45.0 (43.9, 46.1)	44.9 (43.9, 46.0)	38.3 (37.4, 39.2)	47.1 (46.1, 48.1)	51.8 (50.7, 52.9)	54.7 (53.6, 55.8)	45.6 (44.7, 46.6)	35.5 (34.5, 36.6)	45.1 (44.7, 45.4)
Male sex	952 (58.1)	706 (48.9)	687 (47.7)	1272 (77.4)	756 (46.5)	317 (24.1)	598 (36.7)	806 (51.0)	941 (67.0)	2345 (52.0)
Body mass index, kg/m^2^, mean (95%CI)	27.6 (27.2, 28.1)	27.2 (26.8, 27.6)	26.8 (26.4, 27.3)	26.9 (26.6, 27.3)	27.6 (27.2, 27.9)	27.3 (26.8, 27.7)	27.5 (27.1, 27.9)	27.4 (27.0, 27.8)	26.9 (26.4, 27.3)	27.2 (27.0, 27.5)
Weight status										
Underweight/Healthy weight	615 (35.9)	529 (38.6)	577 (40.8)	610 (39.8)	570 (36.8)	541 (38.0)	603 (36.1)	577 (37.1)	541 (41.6)	1721 (38.3)
Overweight/Obesity	1230 (64.1)	1032 (61.4)	904 (59.2)	1121 (60.2)	1119 (63.2)	926 (62.0)	1102 (63.9)	1161 (62.9)	903 (58.4)	3166 (61.7)
Education										
No non-school qualification	771 (41.9)	585 (38.6)	459 (32.1)	642 (39.3)	624 (36.9)	549 (36.9)	637 (37.0)	626 (35.4)	552 (41.0)	1815 (37.8)
Diploma/Certificate	638 (34.5)	555 (34.8)	524 (34.9)	671 (36.6)	589 (35.1)	457 (31.8)	559 (32.8)	620 (36.1)	538 (35.1)	1717 (34.7)
Tertiary	436 (23.5)	421 (26.6)	498 (33.0)	418 (24.1)	476 (27.9)	461 (31.3)	509 (30.1)	492 (28.5)	354 (23.9)	1355 (27.4)
Physical activity ^a^										
Did not meet guidelines	1101 (57.7)	861 (54.8)	738 (49.0)	1001 (56.9)	975 (55.6)	724 (48.5)	898 (53.8)	956 (53.0)	846 (55.4)	2700 (54.1)
Met guidelines	744 (42.3)	700 (45.2)	743 (51.0)	730 (43.1)	714 (44.4)	743 (51.5)	807 (46.2)	782 (47.0)	598 (44.6)	2187 (45.9)
Smoking status										
Current smoker	377 (20.5)	293 (18.1)	181 (11.0)	384 (20.7)	299 (16.0)	168 (12.5)	170 (9.7)	320 (16.3)	361 (23.9)	851 (16.8)
Ex-smoker	601 (28.6)	541 (32.7)	496 (32.3)	531 (25.7)	576 (32.8)	531 (36.1)	684 (39.2)	580 (32.6)	374 (21.9)	1638 (31.0)
Never smoked	867 (50.9)	727 (49.2)	804 (56.8)	816 (53.6)	814 (51.2)	768 (51.4)	851 (51.1)	838 (51.1)	709 (54.2)	2398 (52.2)
Dietary intake										
More than usual	176 (9.7)	152 (8.8)	162 (13.0)	179 (10.4)	183 (10.4)	128 (10.5)	182 (11.4)	183 (10.5)	125 (9.5)	490 (10.5)
Usual	1200 (63.0)	1074 (67.2)	1046 67.0)	1112 (62.5)	1153 (65.9)	1055 (69.5)	1254 (72.8)	1183 (65.6)	883 (59.0)	3320 (65.6)
Less than usual	469 (27.3)	335 (23.9)	273 (20.0)	440 (27.1)	353 (23.7)	284 (20.0)	269 (15.9)	372 (23.9)	436 (31.5)	1077 (23.9)
Misreporting ^b^										
Over-reporting	61 (4.4)	65 (4.7)	99 (5.9)	95 (6.0)	71 (4.4)	59 (4.2)	103 (6.0)	72 (4.9)	50 (4.1)	225 (5.0)
Plausible	1288 (70.2)	1153 (72.3)	1155 (79.3)	1338 (78.7)	1202 (69.3)	1056 (72.1)	1337 (79.5)	1279 (72.7)	980 (69.2)	3596 (73.7)
Under-reporting	496 (25.4)	343 (23.0)	227 (14.9)	298 (15.3)	416 (26.3)	352 (23.7)	265 (14.6)	387 (22.3)	414 (26.7)	1066 (21.4)
Consumed red wine										
No	1502 (82.5)	1292 (84.0)	1228 (84.6)	1390 (83.5)	1386 (83.4)	1246 (84.1)	1396 (81.5)	1414 (82.3)	1212 (86.9)	4022 (83.6)
Yes	343 (17.5)	269 (16.0)	253 (15.4)	341 (16.5)	303 (16.6)	221 (15.9)	309 (18.5)	324 (17.7)	232 (13.1)	865 (16.4)

PDI: plant-based diet index, hPDI: healthful plant-based diet index, uPDI: unhealthful plant-based diet index, CI: confidence interval. *N* values presented are unweighted. Weighted proportion (Weighting factor used: NPAD2WGT for all persons who completed second (CATI) interview) used for proportions (%) and means (95%CIs). ^a^ Guideline of 150 min of physical activity over five sessions in the previous week, ^b^ Ratio of energy intake to predicted total energy expenditure (using sex and age-specific equations based on body weight (kg) and a PAL of “low active” (PAL ≥ 1.4 < 1.6).

**Table 2:** Association between weight status, waist circumference, and blood pressure and plant-based diet indices (PDI, hPDI, and uPDI).

	*n* (%) ^a^	PDI			hPDI			uPDI		
		Mean PDI (95%CI)	Unadjusted OR (95%CI)	Adjusted OR ^b^ (95%CI)	Mean hPDI (95%CI)	Unadjusted OR (95%CI)	Adjusted OR ^b^ (95%CI)	Mean uPDI (95%CI)	Unadjusted OR (95%CI)	Adjusted OR ^b^ (95%CI)
Weight status ^c^										
Underweight/ Normal weight	1721 (38.3%)	54.0 (53.5, 54.4)	Reference	Reference	52.7 (52.1, 53.3)	Reference	Reference	56.0 (55.5, 56.6)	Reference	Reference
Overweight/ Obesity	3166 (61.7%)	53.3 (53.0, 53.6)	0.98 (0.97, 1.00)	1.00 (0.98, 1.01)	52.9 (52.5, 53.2)	1.00 (0.99, 1.02)	0.98 (0.96, 1.00)	55.1 (54.7, 55.5)	0.98 (0.97, 1.00)	1.00 (0.98, 1.02)
Waist circumference ^d^										
Not at risk	1644 (38.1%)	53.7 (53.2, 54.1)	Reference	Reference	51.5 (51.0, 52.1)	Reference	Reference	56.8 (56.3, 57.3)	Reference	Reference
Increased/ Substantially increased risk	3243 (61.9%)	53.5 (53.2, 53.8)	1.00 (0.98, 1.01)	1.01 (0.99, 1.03)	53.6 (53.3, 53.9)	1.03 (1.02, 1.05)	0.98 (0.96, 0.99)	54.6 (54.2, 55.0)	0.96 (0.95, 0.97)	1.00 (0.99, 1.02)
Blood pressure ^e^										
Normal	1942 (42.6%)	54.1 (53.7, 54.4)	Reference	Reference	52.7 (52.3, 53.1)	Reference	Reference	56.2 (55.7, 56.8)	Reference	Reference
High	2945 (57.4%)	53.2 (52.8, 53.6)	0.98 (0.96, 0.99)	0.99 (0.97, 1.00)	52.9 (52.5, 53.2)	1.00 (0.99, 1.01)	0.99 (0.98, 1.01)	54.9 (54.4, 55.3)	0.98 (0.96, 0.99)	1.01 (0.99, 1.03)
Hypertension ^f^										
Not hypertensive	3789 (79.2%)	53.8 (53.5, 54.0)	Reference	Reference	52.6 (52.3, 52.9)	Reference	Reference	55.8 (55.4, 56.1)	Reference	Reference
Hypertensive	1098 (20.8%)	52.9 (52.3, 53.4)	0.98 (0.96, 0.99)	0.98 (0.96, 1.00)	53.5 (52.9, 54.2)	1.02 (1.00, 1.03)	0.99 (0.96, 1.01)	54.3 (53.6, 54.9)	0.97 (0.96, 0.99)	1.02 (1.00, 1.04)

PDI: plant-based diet index, hPDI: healthful plant-based diet index, uPDI: unhealthful plant-based diet index, CI: confidence interval, OR: odds ratio, ^a^ Weighted proportion (Weighting factor used: NPAD2WGT for all persons who completed second (CATI) interview), ^b^ Adjusted for age (continuous), sex, smoking status (categorical: current smoker, ex-smoker, never smoked), physical activity (categorical: Met or not met 150 min five sessions per week guideline), education (categorical: no non-school qualification, certificate/diploma, tertiary), typical diet (categorical: more than usual, usual, less than usual), energy misreporting (plausible, under-reporting, over-reporting), alcohol intake (categorical: non-consumer, consumer) and BMI (continuous), ^c^ Underweight/normal weight: BMI  <  25 kg/m^2^, overweight/obese BMI  ≥  25 kg/m^2^, ^d^ Females: Not at risk < 80 cm, increased/substantially increased risk ≥ 80 cm. Males: Not at risk < 94 cm, increased/substantially increased risk ≥ 94 cm, ^e^ Normal: <120/80 mmHg, high: ≥120/80 mmHg, ^f^ Not hypertensive (<140/90 mmHg), hypertensive (≥140/90 mmHg).

**Table 3:** Association between lipid profiles and diabetes status with the plant-based diet quality indices (PDI, hPDI, and uPDI).

	*n* (%) ^a^	PDI			hPDI			uPDI		
		Mean PDI (95%CI)	Unadjusted OR (95%CI)	Adjusted OR ^b^ (95%CI)	Mean hPDI (95%CI)	Unadjusted OR (95%CI)	Adjusted OR ^b^ (95%CI)	Mean uPDI (95%CI)	Unadjusted OR (95%CI)	Adjusted OR ^b^ (95%CI)
Total cholesterol status ^c^										
Normal	1022 (66.4%)	53.7 (53.1, 54.2)	Reference	Reference	51.9 (51.1, 52.7)	Reference	Reference	56.8 (56.0, 57.6)	Reference	Reference
Abnormal	750 (33.6%)	53.6 (53.0, 54.2)	1.00 (0.97, 1.02)	1.00 (0.98, 1.03)	54.4 (53.4, 55.4)	1.04 (1.02, 1.06)	1.01 (0.99, 1.04)	53.5 (52.6, 54.5)	0.94 (0.92, 0.97)	0.98 (0.95, 1.00)
HDL cholesterol status ^d^										
Normal	1399 (79.3%)	53.6 (53.1, 54.0)	Reference	Reference	52.8 (52.1, 53.5)	Reference	Reference	55.6 (54.9, 56.4)	Reference	Reference
Abnormal	373 (20.7%)	53.9 (52.9, 54.9)	1.01 (0.98, 1.04)	1.01 (0.98, 1.04)	52.5 (51.2, 53.9)	1.00 (0.97, 1.02)	0.97 (0.94, 1.00)	55.9 (55.0, 56.9)	1.01 (0.98, 1.03)	1.01 (0.98, 1.04)
Fasting LDL cholesterol status ^e^										
Normal	1032 (65.1%)	53.6 (53.1, 54.2)	Reference	Reference	52.1 (51.4, 52.8)	Reference	Reference	56.7 (55.9, 57.5)	Reference	Reference
Abnormal	740 (34.9%)	53.7 (53.0, 54.3)	1.00 (0.98, 1.03)	1.01 (0.98, 1.04)	53.9 (52.9, 54.9)	1.03 (1.01, 1.05)	1.01 (0.98, 1.04)	53.8 (52.9, 54.7)	0.95 (0.93, 0.97)	0.98 (0.96, 1.01)
Fasting triglycerides status ^f^										
Normal	1543 (88.9%)	53.7 (53.2, 54.1)	Reference	Reference	52.8 (52.2, 53.5)	Reference	Reference	55.7 (55.0, 56.3)	Reference	Reference
Abnormal	229 (11.1%)	53.5 (52.3, 54.7)	0.99 (0.96, 1.03)	1.02 (0.97, 1.06)	51.9 (49.9, 53.9)	0.98 (0.95, 1.02)	1.00 (0.96, 1.04)	56.0 (55.0, 56.9)	1.01 (0.98, 1.03)	1.00 (0.97, 1.04)
Dyslipidaemia ^g^										
No	671 (45.2%)	53.5 (52.7, 54.2)	Reference	Reference	51.9 (50.9, 52.9)	Reference	Reference	57.3 (56.2, 58.3)	Reference	Reference
Yes	1101 (54.8%)	53.8 (53.2, 54.4)	1.01 (0.98, 1.04)	1.01 (0.98, 1.05)	53.4 (52.6, 54.2)	1.03 (1.00, 1.05)	1.00 (0.96, 1.03)	54.4 (53.7, 55.1)	0.95 (0.93, 0.97)	0.98 (0.95, 1.01)
Diabetes status (based on glucose) ^h^										
No	1690 (96.4%)	53.7 (53.3, 54.0)	Reference	Reference	52.7 (52.1, 53.3)	Reference	Reference	55.7 (55.1, 56.3)	Reference	Reference
Yes or impaired fasting plasma glucose	82 (3.6%)	53.1 (50.7, 55.6)	0.98 (0.92, 1.06)	1.00 (0.94, 1.07)	52.6 (49.4, 55.8)	1.00 (0.94, 1.05)	1.00 (0.94, 1.07)	54.5 (52.5, 56.6)	0.98 (0.94, 1.02)	1.01 (0.95, 1.08)
Diabetes status (based on HbA1c) ^i^										
No	1674 (95.0%)	53.7 (53.3, 54.1)	Reference	Reference	52.6 (52.0, 53.2)	Reference	Reference	55.8 (55.2, 56.4)	Reference	Reference
Yes or at risk of diabetes	98 (5.0%)	53.0 (50.8, 55.2)	0.98 (0.92, 1.04)	0.99 (0.93, 1.05)	54.7 (52.7, 56.7)	1.03 (1.00, 1.07)	1.02 (0.97, 1.07)	53.5 (51.9, 55.1)	0.96 (0.93, 0.99)	1.01 (0.97, 1.06)

PDI: plant-based diet index, hPDI: healthful plant-based diet index, uPDI: unhealthful plant-based diet index, CI: confidence interval, OR: odds ratio, HDL: high density lipoprotein, LDL: low density lipoprotein, ^a^ Weighted proportion (Weighting factor used: NHMSPERW for biomedical participants), ^b^ Adjusted for age (continuous), sex, smoking status (categorical: current smoker, ex-smoker, never smoked), physical activity (categorical: Met or Not met 150 min five sessions per week guideline), education (categorical: no non-school qualification, certificate/diploma, tertiary), typical diet (categorical: more than usual, usual, less than usual), energy misreporting (plausible, under-reporting, over-reporting), alcohol intake (categorical: non-consumer, consumer). For blood pressure, additionally adjusted for BMI (continuous) and known hypertension (categorical: condition known and current, condition not known or not current), ^c^ Normal cholesterol status: <5.5 mmol/L; Abnormal cholesterol status: ≥5.5 mmol/L, ^d^ Normal HDL status: Males ≥ 1.0 mmol/L or Females ≥ 1.3 mmol/L; Abnormal HDL status: Males < 1.0 mmol/L or Females < 1.3 mmol/L; Abnormal, ^e^ Normal LDL status: <3.5 mmol/L; Abnormal; Abnormal LDL status: ≥3.5 mmol/L (Persons with fasting triglycerides results ≥ 4.5 mmol/L excluded), ^f^ Normal triglycerides status: <2.0 mmol/L; Abnormal triglycerides status: ≥2.0 mmol/L, ^g^ No dyslipidaemia: Considered ‘Normal’ for cholesterol, HDL (sex-dependent), LDL and triglycerides; Dyslipidaemia: Considered ‘Abnormal’ for cholesterol, HDL (sex-dependent), LDL or triglycerides, ^h^ No diabetes: Fasting plasma glucose ≤ 6.0 mmol/L; Impaired fasting plasma glucose or has diabetes: Fasting plasma glucose > 6.0 mmol/L, ^i^ No diabetes: HbA1c < 6.0%; At risk of diabetes or has diabetes: HbA1c ≥ 6.0%.

## Data Availability

Restrictions apply to the availability of these data. Data were obtained from Australian Bureau of Statistics and are available at https://www.abs.gov.au/about/data-services/consultancy-services#whereto-find-abs-data (accessed on 3 March 2020) with the permission of Australian Bureau of Statistics.

## Supplementary Materials

The following supporting information can be downloaded at: https://www.mdpi.com/article/10.3390/nu17101621/s1, Table S1: STROBE-nut: An extension of the STROBE statement for nutritional epidemiology; Table S2: Characteristics of included and excluded adult study participants (*n* = 9435 adults 18 years and over); Table S3: Categorisation of all major and sub-major food groups in AUSNUT, and their corresponding classification to the 18 food groups in the three plant-based diet quality indices; Figure S1: Directed acyclic graph (DAG) for identification of confounders for statistical analysis.

## Abbreviations

The following abbreviations are used in this manuscript:

AHS	Australian Health Survey
AUSNUT	Australian food and nutrient database
BMI	Body mass index
CATI	Computer assisted telephone interviewing
CVD	Cardiovascular disease
DAG	Directed acyclic graph
DASH	Dietary Approaches to Stop Hypertension
DGI	Dietary Guideline Index
HbA1C	Haemoglobin A1C
HDL	High-density lipoprotein
hPDI	Healthful plant-based-diet index
LDL	Low-density lipoprotein
MSM	Multiple source method
NNPAS	National Nutrition and Physical Activity Survey
OR	Odds ratio
PAL	Physical activity level
PDI	Overall plant-based diet index
STROBE-nut	Strengthening the Reporting of Observational Studies in Epidemiology–nutritional epidemiology
uPDI	Unhealthful plant-based diet index
