# Supplementary material for: Plant-Based Diet Quality Is Associated with Cardiometabolic Health in Adults: A Cross-Sectional Analysis of the Australian Health Survey
Source: Nutrients. 2025 May 9;17(10):1621. doi: 10.3390/nu17101621 (PMC12113780; doi:10.3390/nu17101621)

Title: Plant-based diet quality is associated with cardiometabolic health in adults: a cross-sectional analysis of the Australian Health Survey

Authors: Kacie M Dickinson, Laura E Marchese, Katherine M Livingstone

Supplementary Material

Supplemental Table S1: STROBE-nut: An extension of the STROBE statement for nutritional epidemiology

Item	Item nr	STROBE recommendations	Extension for Nutritional Epidemiology studies (STROBE-nut)	Reported on page #
**Title and abstract**	1	(a) Indicate the study's design with a commonly used term in the title or the abstract.<br>(b) Provide in the abstract an informative and balanced summary of what was done and what was found.	**nut-1** State the dietary/nutritional assessment method(s) used in the title, abstract, or keywords.	**1**
**Introduction**				**2**
Background rationale	2	Explain the scientific background and rationale for the investigation being reported.		2
Objectives	3	State specific objectives, including any pre-specified hypotheses.		2
**Methods**				3-6
Study design	4	Present key elements of study design early in the paper.		3
Settings	5	Describe the setting, locations, and relevant dates, including periods of recruitment, exposure, follow-up, and data collection.	**nut-5** Describe any characteristics of the study settings that might affect the dietary intake or nutritional status of the participants, if applicable.	3
Participants	6	a) Cohort study—Give the eligibility criteria, and the sources and methods of selection of participants. Describe methods of follow-up.<br>Case-control study—Give the eligibility criteria, and the sources and methods of case ascertainment and control selection. Give the rationale for the choice of cases and controls.	**nut-6** Report particular dietary, physiological or nutritional characteristics that were considered when selecting the target population.	3

Title: Plant-based diet quality is associated with cardiometabolic health in adults: a cross-sectional analysis of the Australian Health Survey

Authors: Kacie M Dickinson, Laura E Marchese, Katherine M Livingstone

Item	Item nr	STROBE recommendations	Extension for Nutritional Epidemiology studies (STROBE-nut)	Reported on page #
		Cross-sectional study—Give the eligibility criteria, and the sources and methods of selection of participants.<br>(b) Cohort study—For matched studies, give matching criteria and number of exposed and unexposed.<br>Case-control study—For matched studies, give matching criteria and the number of controls per case.		
Variables	7	Clearly define all outcomes, exposures, predictors, potential confounders, and effect modifiers. Give diagnostic criteria, if applicable.	**nut-7.1** Clearly define foods, food groups, nutrients, or other food components.<br>**nut-7.2** When using dietary patterns or indices, describe the methods to obtain them and their nutritional properties.	3-4
Data sources - measurements	8	For each variable of interest, give sources of data and details of methods of assessment (measurement).Describe comparability of assessment methods if there is more than one group.	**nut-8.1** Describe the dietary assessment method(s), e.g., portion size estimation, number of days and items recorded, how it was developed and administered, and how quality was assured. Report if and how supplement intake was assessed.<br>**nut-8.2** Describe and justify food composition data used. Explain the procedure to match food composition with consumption data. Describe the use of conversion factors, if applicable.<br>**nut-8.3** Describe the nutrient requirements, recommendations, or dietary guidelines and the evaluation approach used to compare intake with the dietary reference values, if applicable.<br>**nut-8.4** When using nutritional biomarkers, additionally use the STROBE Extension for Molecular Epidemiology (STROBE-ME). Report the type of biomarkers used and their usefulness as dietary exposure markers.<br>**nut-8.5** Describe the assessment of nondietary data (e.g., nutritional status and influencing factors) and timing of the assessment of these variables in relation to dietary assessment.	3-4

Title: Plant-based diet quality is associated with cardiometabolic health in adults: a cross-sectional analysis of the Australian Health Survey

Authors: Kacie M Dickinson, Laura E Marchese, Katherine M Livingstone

Item	Item nr	STROBE recommendations	Extension for Nutritional Epidemiology studies (STROBE-nut)	Reported on page #
			**nut-8.6** Report on the validity of the dietary or nutritional assessment methods and any internal or external validation used in the study, if applicable.	
Bias	9	Describe any efforts to address potential sources of bias.	**nut-9** Report how bias in dietary or nutritional assessment was addressed, e.g., misreporting, changes in habits as a result of being measured, or data imputation from other sources	5
Study Size	10	Explain how the study size was arrived at.		Figure 1
Quantitative variables	11	Explain how quantitative variables were handled in the analyses. If applicable, describe which groupings were chosen and why.	**nut-11** Explain categorization of dietary/nutritional data (e.g., use of N-tiles and handling of nonconsumers) and the choice of reference category, if applicable.	6
Statistical Methods	12	(a) Describe all statistical methods, including those used to control for confounding<br>(b) Describe any methods used to examine subgroups and interactions.<br>(c) Explain how missing data were addressed.<br>(d) Cohort study—If applicable, explain how loss to follow-up was addressed.<br>Case-control study—If applicable, explain how matching of cases and controls was addressed.<br>Cross-sectional study—If applicable, describe analytical methods taking account of sampling strategy.<br>(e) Describe any sensitivity analyses.	**nut-12.1** Describe any statistical method used to combine dietary or nutritional data, if applicable.<br>**nut-12.2** Describe and justify the method for energy adjustments, intake modeling, and use of weighting factors, if applicable.<br>**nut-12.3** Report any adjustments for measurement error, i.e,. from a validity or calibration study.	5-6
**Results**				**6-13**
Participants	13	(a) Report the numbers of individuals at each stage of the study—e.g., numbers potentially eligible, examined for eligibility, confirmed eligible, included in the study, completing follow-up, and analyzed.<br>(b) Give reasons for non-participation at each stage.<br>(c) Consider use of a flow diagram.	**nut-13** Report the number of individuals excluded based on missing, incomplete or implausible dietary/nutritional data.	Figure 1

Title: Plant-based diet quality is associated with cardiometabolic health in adults: a cross-sectional analysis of the Australian Health Survey

Authors: Kacie M Dickinson, Laura E Marchese, Katherine M Livingstone

Item	Item nr	STROBE recommendations	Extension for Nutritional Epidemiology studies (STROBE-nut)	Reported on page #
Descriptive data	14	(a) Give characteristics of study participants (e.g., demographic, clinical, social) and information on exposures and potential confounders<br>(b) Indicate the number of participants with missing data for each variable of interest<br>(c) Cohort study—Summarize follow-up time (e.g., average and total amount)	**nut-14** Give the distribution of participant characteristics across the exposure variables if applicable. Specify if food consumption of total population or consumers only were used to obtain results.	Table 1
Outcome data	15	Cohort study—Report numbers of outcome events or summary measures over time.<br>Case-control study—Report numbers in each exposure category, or summary measures of exposure.<br>Cross-sectional study—Report numbers of outcome events or summary measures.		Tables 2 and 3
Main results	16	(14) Give unadjusted estimates and, if applicable, confounder-adjusted estimates and their precision (e.g., 95% confidence interval).<br>Make clear which confounders were adjusted for and why they were included.<br>(b) Report category boundaries when continuous variables were categorized.<br>© If relevant, consider translating estimates of relative risk into absolute risk for a meaningful time period.	**Nut-16** Specify if nutrient intakes are reported with or without inclusion of dietary supplement intake, if applicable.	Not applicable
Other analyses	17	Report other analyses done—e.g., analyses of subgroups and interactions and sensitivity analyses.	**nut-17** Report any sensitivity analysis (e.g., exclusion of misreporters or outliers) and data imputation, if applicable.	Not applicable
**Discussion**				13-15
Key results	18	Summarize key results with reference to study objectives.		13
Limitation	19	Discuss limitations of the study, taking into account sources of potential bias or imprecision. Discuss both direction and magnitude of any potential bias.	**nut-19** Describe the main limitations of the data sources and assessment methods used and implications for the interpretation of the findings.	15

Title: Plant-based diet quality is associated with cardiometabolic health in adults: a cross-sectional analysis of the Australian Health Survey

Authors: Kacie M Dickinson, Laura E Marchese, Katherine M Livingstone

Item	Item nr	STROBE recommendations	Extension for Nutritional Epidemiology studies (STROBE-nut)	Reported on page #
Interpretation	20	Give a cautious overall interpretation of results considering objectives, limitations, multiplicity of analyses, results from similar studies, and other relevant evidence.	**nut-20** Report the nutritional relevance of the findings, given the complexity of diet or nutrition as an exposure.	13-15
Generalizability	21	Discuss the generalizability (external validity) of the study results.		13-15
**Other information**				
Funding	22	Give the source of funding and the role of the funders for the present study and, if applicable, for the original study on which the present article is based.		16
Ethics			**nut-22.1** Describe the procedure for consent and study approval from ethics committee(s).	5
Supplementary material			**nut-22.2** Provide data collection tools and data as online material or explain how they can be accessed.	Separate document

Title: Plant-based diet quality is associated with cardiometabolic health in adults: a cross-sectional analysis of the Australian Health Survey

Authors: Kacie M Dickinson, Laura E Marchese, Katherine M Livingstone

Supplementary Table S2: Characteristics of included and excluded adult study participants (n=9435 adults 18 years and over)

**Characteristics**	**Included in primary analysis (n=4,887)**	**Excluded from primary analysis (n=4,548)**
Male	2345 (52.2)	1984 (46.3)
Female	2542 (47.8)	2564 (53.7)
Age in years, mean (95% CI)	45.6 (45.1, 46.0)	46.0 (45.6, 46.5)
Age group		
18–44	2141 (49.5)	2099 (50.6)
45–64	1672 (33.6)	1420 (32.0)
65–84	1074 (16.9)	841 (14.1)
85 and over	0 (0.0)	188 (3.2)
Recall day		
1 day	0 (0.0)	3321 (75.3)
2 days	4887 (100.0)	1227 (24.7)
Education		
No non-school qualification	1815 (37.7)	1889 (40.0)
Diploma/Certificate	1717 (34.8)	1485 (34.8)
Tertiary	1355 (27.5)	1030 (22.3)
Level not determined	0 (0.0)	144 (3.0)
Physical activity		
Did not meet guidelines	2700 (53.6)	2723 (60.0)
Met guidelines	2187 (46.4)	1736 (38.0)
Not known	0 (0.0)	89 (2.0)
Smoking status		
Current smoker	851 (16.5)	934 (18.8)
Ex-smoker	1638 (31.6)	1439 (30.2)
Never smoked	2398 (51.9)	2175 (51.1)

N values presented are unweighted. Weighted proportion (Weighting factor used: NPAFINWT for all persons) used for proportions (%) and means (95% CIs). All values are n (%) unless otherwise specified.

Title: Plant-based diet quality is associated with cardiometabolic health in adults: a cross-sectional analysis of the Australian Health Survey

Authors: Kacie M Dickinson, Laura E Marchese, Katherine M Livingstone

Supplementary Table S3: Categorisation of all major and sub-major food groups in AUSNUT, and their corresponding classification to the 18 food groups in the three plant-based diet quality indices

2011-13 Major food group code	2011-13 Sub-major food group code	2011-13 Sub-major food group name	Classification according to Satija paper	
11 Non-alcoholic beverages	111	Tea	7	Tea and Coffee
	112	Coffee and coffee substitutes	7	Tea and Coffee
	113	Fruit and vegetable juices, and drinks	2	Fruits for freshly-squeezed fruit juices
			3	Vegetables for all vegetable juices
			8	Fruit juices for all other fruit juices including fruit and vegetable juice blends
			11	Sugar sweetened beverages for all fruit drinks including ready to drink or made from concentrate or from dry powder
	114	Cordials	11	Sugar sweetened beverages
			19	Excluded if intense sweetened
	115	Soft drinks, and flavoured mineral waters	11	Sugar sweetened beverages
			19	Excluded if intense sweetened
	116	Electrolyte, energy and fortified drinks	11	Sugar sweetened beverages
			19	Excluded if intense sweetened
	117	Waters, municipal and bottled, unflavoured	19	Excluded from analysis
	118	Other beverage flavourings and prepared beverages	14	Dairy – mainly Milo, hot chocolate in this category
12 Cereals and cereal products	121	Flours and other cereal grains and starches	1	Grains: Barley, buckwheat, bulgur, grains, millet, oat bran, oats, quinoa, wild rice, rye, spelt, wheat bran<br>Rice: Brown rice and red rice<br>Cereal flours: Rye, spelt, wheat wholemeal
			9	Grains: Cornmeal, sago<br>Rice: White rice<br>Cereal flours: Couscous, arrowroot, cornflour, gluten free mix, rice, wheat white, gluten, rice paper wrapper, semolina, tapioca and all fortified cereal flours and starches

Title: Plant-based diet quality is associated with cardiometabolic health in adults: a cross-sectional analysis of the Australian Health Survey

Authors: Kacie M Dickinson, Laura E Marchese, Katherine M Livingstone

2011-13 Major food group code	2011-13 Sub-major food group code	2011-13 Sub-major food group name	Classification according to Satija paper	
	122	Regular breads, and bread rolls (plain/unfilled/untopped varieties)	1	Mixed grain, wholemeal and brown, rye, gluten free with added grains
			9	White breads and rolls, gluten free including not stated as to major flour or fortification (Component higher in refined grains than whole grains)
	123	English-style muffins, flat breads, and savoury and sweet breads	1	English-style muffins made with wholemeal flour; Flat bread made with wholemeal or mixed grain; French toast made with wholemeal bread
			9	All other English-style muffins; all other flat bread; all savoury filled or topped breads and rolls; all sweet breads and buns and fried bread; French toast made with white bread, garlic bread (commercial and homemade)
	124	Pasta and pasta products (without sauce)	1	Pasta made with wholemeal wheat; buckwheat noodle
			9	All other pasta and noodles; all instant noodles and products; rice stick noodles, gluten free pasta, maize flour pasta; filled pasta (some have vegetable/ cheese/ meat fillings but refined grains is major component)
	125	Breakfast cereals, ready to eat	1	Breakfast cereals containing >5% dietary fibre
			9	Breakfast cereals containing ≤5% dietary fibre; all puffed or popped rice breakfast cereals; some mixed grains with rice & wheat only
	126	Breakfast cereals, hot porridge style	1	Oat porridge, brown rice porridge
			9	Rice porridge
			14	Paste, flour mixed with sugar & cow's milk (contains 77% dairy)
13	131	Sweet biscuits	9	Refined grains
	132	Savoury biscuits	1	Biscuits from wholemeal wheat flour with added grains, high fibre,

Title: Plant-based diet quality is associated with cardiometabolic health in adults: a cross-sectional analysis of the Australian Health Survey

Authors: Kacie M Dickinson, Laura E Marchese, Katherine M Livingstone

2011-13 Major food group code	2011-13 Sub-major food group code	2011-13 Sub-major food group name	Classification according to Satija paper	
Cereal based products and dishes				wholemeal wheat flour & rye flour, multigrain corn cake
			9	Biscuits from wheat flour, or white wheat flour, or wholemeal wheat flour, or corn (not multigrain) or not specified
	133	Cakes, muffins, scones, cake-type desserts	9	Refined grains
	134	Pastries	9	Refined grains
	135	Mixed dishes where cereal is the major ingredient	1	Brown rice with no meat
			9	Sandwiches, bread rolls, taco with no meat; wraps with no meat or not further defined; Pasta with no meat; Rice with no meat (except brown rice); noodles with no meat or not further defined; Steamed bun with no meat; dumpling with no meat; Sushi with no meat; Couscous with no meat; Polenta with no meat
			10	Gnocchi with no meat
			16	Sandwiches, bread rolls, taco, wraps filled with tuna; Pasta with seafood; Rice or risotto with seafood; Sushi with fish or seafood or not further defined;
			17	Sandwiches, bread rolls filled with meat (ham, pork, bacon, frankfurt, chicken, beef; +/- salads, +/- cheese) or not further defined; wraps, taco with meat (ham, pork, bacon, frankfurt, chicken, beef; +/- salads, +/- cheese); Pasta with meat or not further defined; Rice or risotto with meat; Gnocchi with meat; Steamed bun with meat; Sushi with meat; Couscous with meat
			18	Pizza
	136	Batter-based products	1	Crumpet made from wholemeal flour
			9	Refined grains
14	141	Butters	13	Animal fat

Title: Plant-based diet quality is associated with cardiometabolic health in adults: a cross-sectional analysis of the Australian Health Survey

Authors: Kacie M Dickinson, Laura E Marchese, Katherine M Livingstone

2011-13 Major food group code	2011-13 Sub-major food group code	2011-13 Sub-major food group name	Classification according to Satija paper	
Fats and oils	142	Dairy blends	13	Animal fat
	143	Margarine and table spreads	6	Vegetable oils
	144	Plant oils	6	Vegetable oils
	145	Other fats	6	Vegetable-based solid fats
			13	Animal-based solid fats or blend of animal and vegetable fats
	146	Unspecified fats	6	Oil
			13	Fat, butter, dairy blend or margarine spread; other fat or oil, not further specified
15 Fish and seafood products and dishes	151	Fin fish (excluding commercially sterile)	16	Fish or seafood
	152	Crustacea and molluscs (excluding commercially sterile)	16	Fish or seafood
	153	Other sea and freshwater foods	16	Fish or seafood
	154	Packed (commercially sterile) fish and seafood	16	Fish or seafood
	155	Fish and seafood products (homemade and takeaway)	16	Fish or seafood
	156	Mixed dishes with fish or seafood as the major component	16	Fish or seafood
16 Fruit products and dishes	161	Pome fruit	2	Fruits
	162	Berry fruit	2	Fruits
	163	Citrus fruit	2	Fruits
	164	Stone fruit	2	Fruits
	165	Tropical and subtropical fruit	2	Fruits
	167	Mixtures of two or more groups of fruit	2	Fruits
	168	Dried fruit, preserved fruit	2	Fruits
	169	Mixed dishes where fruit is the major component	2	Fruits
17 Egg products and dishes	171	Eggs	15	Egg
	172	Dishes where egg is the major ingredient	15	Egg

Title: Plant-based diet quality is associated with cardiometabolic health in adults: a cross-sectional analysis of the Australian Health Survey

Authors: Kacie M Dickinson, Laura E Marchese, Katherine M Livingstone

2011-13 Major food group code	2011-13 Sub-major food group code	2011-13 Sub-major food group name	Classification according to Satija paper	
18<br>Meat poultry and game products and dishes	181	Beef, sheep and pork, unprocessed	17	Meat
	182	Mammalian game meats	17	Meat
	183	Poultry and feathered game	17	Meat
	184	Organ meats and offal, products and dishes	17	Meat
	185	Sausages, frankfurts and saveloys	17	Meat
	186	Processed meat	17	Meat
	187	Mixed dishes where beef, sheep, pork or mammalian game is the major component	17	Meat
	188	Mixed dishes where sausage, bacon, ham or other processed meat is the major component	17	Meat
	189	Mixed dishes where poultry or feathered game is the major component	17	Meat
19<br>Milk products and dishes	191	Dairy milk (cow, sheep and goat)	14	Dairy
	192	Yoghurt	14	Dairy
	193	Cream	14	Dairy
	194	Cheese	14	Dairy
	195	Frozen milk products	14	Dairy
	196	Custards	14	Dairy
	197	Other dishes where milk or a milk product is the major component	14	Dairy
	198	Flavoured milks and milkshakes	14	Dairy
20<br>Dairy and meat substitutes	201	Dairy milk substitutes, unflavoured	5	Legumes
	202	Dairy milk substitutes, flavoured	5	Legumes
	203	Cheese substitute	5	Legumes
	204	Soy-based ice confection	5	Legumes
	205	Soy-based yoghurts	5	Legumes
	206	Meat substitutes	5	Legumes

Title: Plant-based diet quality is associated with cardiometabolic health in adults: a cross-sectional analysis of the Australian Health Survey

Authors: Kacie M Dickinson, Laura E Marchese, Katherine M Livingstone

2011-13 Major food group code	2011-13 Sub-major food group code	2011-13 Sub-major food group name	Classification according to Satija paper	
	207	Dishes where meat substitutes are the major component	5	Legumes
21 Soup	211	Soup, homemade from basic ingredients	3	Vegetable soup (not creamy)
			18	Soup containing meat, poultry or seafood; Vegetable soup with cream
	212	Dry soup mix	3	Vegetable soup (not creamy)
			18	Soup containing meat, poultry or seafood; Vegetable soup with cream
	213	Soup, prepared from dry soup mix	3	Vegetable soup (not creamy)
			18	Soup containing meat, poultry or seafood; Vegetable soup with cream
	214	Canned condensed soup (unprepared)	3	Vegetable soup (not creamy)
			18	Soup containing meat, poultry or seafood; Vegetable soup with cream
	215	Soup, commercially sterile, prepared from condensed or sold ready to heat	3	Vegetable soup (not creamy)
			18	Soup containing meat, poultry or seafood; Vegetable soup with cream
	216	Soup, not commercially sterile, purchased ready to eat	3	Vegetable soup (not creamy)
			18	Soup containing meat, poultry or seafood; Vegetable soup with cream
22 Seed and nut products and dishes	221	Seeds and seed products	4	Nuts
	222	Nuts and nut products	4	Nuts
23 Savoury sauces and condiments	231	Gravies and savoury sauces	3	Tomato based savoury sauces, homemade or commercial
			18	Gravies (prepared or dry mixes); Non-tomato based savoury sauces
	232	Pickles, chutneys and relishes	2	Fruit-based pickles, chutneys and relishes
			3	Vegetable-based pickles, chutneys and relishes
	233	Salad dressings	6	Italian and French-style dressings
			18	Mayonnaise and cream-style dressings
			19	Excluded

Title: Plant-based diet quality is associated with cardiometabolic health in adults: a cross-sectional analysis of the Australian Health Survey

Authors: Kacie M Dickinson, Laura E Marchese, Katherine M Livingstone

2011-13 Major food group code	2011-13 Sub-major food group code	2011-13 Sub-major food group name	Classification according to Satija paper	
	234	Stuffings	9	Refined grains
	235	Dips	3	Vegetable based dips
			5	Legume based dips
			14	Dairy based dips; Dips not further defined
			16	Taramosalata dip
24 Vegetable products and dishes	241	Potatoes	10	Potato
	242	Cabbage, cauliflower and similar brassica vegetables	3	Vegetables
	243	Carrot and similar root vegetables	3	Vegetables
	244	Leaf and stalk vegetables	3	Vegetables
	245	Peas and beans	3	Vegetables
	246	Tomato and tomato products	3	Vegetables
	247	Other fruiting vegetables	3	Vegetables
	248	Other vegetables and vegetable combinations	3	Vegetables
	249	Dishes where vegetable is the major component	3	Vegetable dishes with no meat; dishes not further defined
			10	Potato salad
			16	Dishes with fish or seafood
			17	Dishes with meat
25 Legume and pulse products and dishes	251	Mature legumes and pulses	5	Legumes
	252	Mature legume and pulse products and dishes	5	Legumes
26 Snack foods	261	Potato snacks	10	Potato crisps or chips or straws; beetroot crisps or chips; vegetable crisps or chips
	262	Corn snacks	1	Popcorn
			10	Corn chips, taco shell
	263	Extruded or reformed snacks	9	Refined grains
	264	Other snacks	5	Pappadams; Bhuja snack mix
			9	Savoury crackers/ biscuits without cheese
			14	Savoury crackers/ biscuits with cheese

Title: Plant-based diet quality is associated with cardiometabolic health in adults: a cross-sectional analysis of the Australian Health Survey

Authors: Kacie M Dickinson, Laura E Marchese, Katherine M Livingstone

2011-13 Major food group code	2011-13 Sub-major food group code	2011-13 Sub-major food group name	Classification according to Satija paper	
			16	Prawn crackers
			17	Pork rind snack
27 Sugar products and dishes	271	Sugar, honey and syrups	12	Sweets and Desserts
	272	Jam and lemon spreads, chocolate spreads, sauces	12	Sweets and Desserts
	273	Dishes and products other than confectionery where sugar is the major component	12	Sweets and Desserts
28 Confectionery and cereal/nut/fruit/seed bars	281	Chocolate and chocolate-based confectionery	12	Sweets and Desserts
	282	Fruit, nut and seed-bars	12	Sweets and Desserts
	283	Muesli or cereal style bars	12	Sweets and Desserts
	284	Other confectionery	12	Sweets and Desserts
			19	Excluded if intense sweetened
29 Alcoholic beverages	291	Beers	19	Excluded
	292	Wines	19	Excluded
	293	Spirits	19	Excluded
	294	Cider and perry	19	Excluded
	295	Other alcoholic beverages	19	Excluded
30 Special dietary foods	301	Formula dietary foods	11	Energy gel; Beverage water based
			12	Meal replacement bar
			14	Meal replacement drink; Very low energy diet drink; Protein drink or powder; Oral supplement beverage
31 Miscellaneous	311	Yeast, and yeast vegetable or meat extracts	19	Excluded
	312	Intense sweetening agents	19	Excluded
	313	Herbs, spices, seasonings and stock cubes	19	Excluded
	314	Essences	19	Excluded
	315	Chemical raising agents and cooking ingredients	19	Excluded

Title: Plant-based diet quality is associated with cardiometabolic health in adults: a cross-sectional analysis of the Australian Health Survey

Authors: Kacie M Dickinson, Laura E Marchese, Katherine M Livingstone

**2011-13 Major food group code**	**2011-13 Sub-major food group code**	**2011-13 Sub-major food group name**	**Classification according to Satija paper**	
32 Infant formulae and foods	321	Infant formulae and human breast milk	19	Excluded
	322	Infant cereal products	19	Excluded
	323	Infant foods	19	Excluded
	324	Infant drinks	19	Excluded
33 Dietary supplements	331	Vitamin and/or mineral supplements	19	Excluded
	332	Oil supplements	19	Excluded
	333	Herbal and homoeopathic supplements	19	Excluded
	334	Other nutritive supplements	19	Excluded
	335	Other non-nutritive supplements	19	Excluded
	336	Other medicines	19	Excluded

Title: Plant-based diet quality is associated with cardiometabolic health in adults: a cross-sectional analysis of the Australian Health Survey

Authors: Kacie M Dickinson, Laura E Marchese, Katherine M Livingstone

Supplementary Figure S1: Directed acyclic graph (DAG) for identification of confounders for statistical analysis

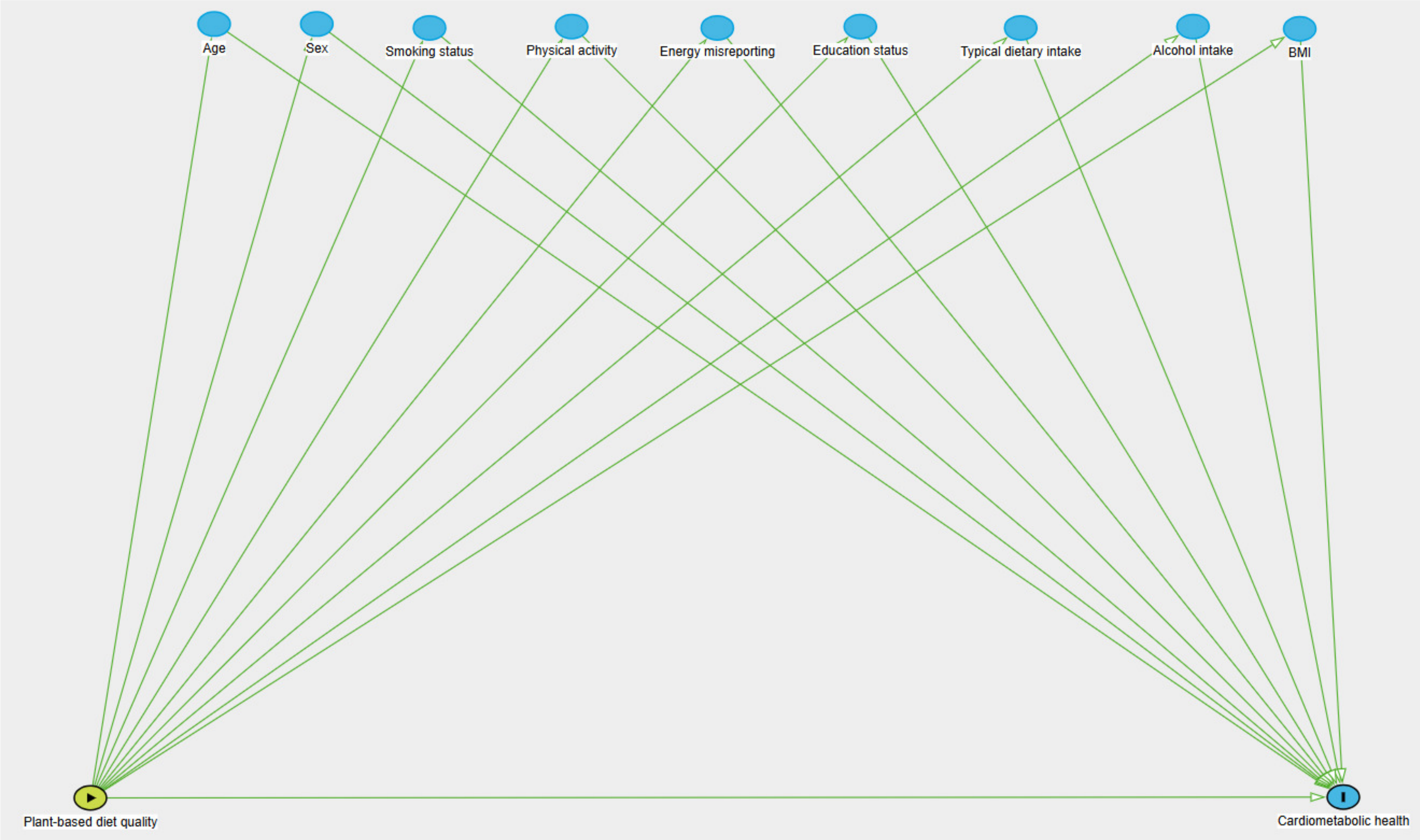

Supplement: Supplementary file 1 [file nutrients-17-01621-s001.zip › nutrients-3614633-supplementary.pdf]
